# Evaluation of Silymarin for management of anti-tuberculosis drug induced liver injury: a randomized clinical trial 

**Published:** 2019

**Authors:** Majid Marjani, Fanak Fahim, Makan Sadr, Mehdi Kazempour Dizaji, Afshin Moniri, Shadi Khabiri, Payam Tabarsi, Ali Akbar Velayati

**Affiliations:** 1 *Clinical Tuberculosis and Epidemiology Research Center, National Research Institute of Tuberculosis and Lung Diseases (NRITLD), Masih Daneshvari Hospital, Shahid Beheshti University of Medical Sciences, Tehran, Iran *; 2 *Department of Clinical Pharmacy, School of Pharmacy, Shahid Beheshti University of Medical Sciences, Tehran, Iran.*; 3 *Virology Research Center, National Research Institute of Tuberculosis and Lung Diseases (NRITLD), Masih Daneshvari Hospital, Shahid Beheshti University of Medical Sciences, Tehran, Iran*; 4 *Mycobacteriology Research Center, National Research Institute of Tuberculosis and Lung Diseases (NRITLD), Masih Daneshvari Hospital, Shahid Beheshti University of Medical Sciences, Tehran, Iran *

**Keywords:** Tuberculosis, Adverse effects, Silymarin, Drug induced hepatitis

## Abstract

**Aim:**

This study was performed to evaluate the potential efficacy of silymarin in the management of anti-tuberculosis medication’s induced liver injury.

**Background:**

Hepatic toxicity is the most serious complication in treatment of tuberculosis.

**Methods:**

In a randomized double blind clinical trial (ACTRN12610000643077), 55 cases with hepatotoxicity caused by anti-tuberculosis drugs were divided into two groups. Informed consents were obtained. The intervention group received silymarin and the control group received placebo. Severity of liver injury, the duration necessary for normalization of liver function and hospital stay were compared between the two groups.

**Results:**

There was not any statistically significant difference in the rate of adverse effects between silymarin and placebo groups.

**Conclusion:**

Although silymarin is considered a safe herbal medication, it was not effective to treat hepatic toxicity of anti-tuberculosis drugs.

## Introduction

 World health organization estimated the incidence of tuberculosis (TB) as 10.4 million in the year 2016 (1). Classic treatment of TB is a combination therapy consisting three hepatotoxic drugs: isoniazid, rifampin, and pyrazinamide. Drug induced liver injury (DILI) is the most serious adverse effect of TB treatment with occurring in 2-28% of the patients receiving anti-TBs. (2, 3) and results in a considerable morbidities or mortalities (4). Adverse effects decrease patient’s adherence and treatment effectiveness, indirectly may contribute to treatment failure or the emergence of drug resistance (5, 6). DILI leads to temporal interruption of TB treatment and only after recovery of liver function; restart of basic drugs is possible. So any intervention that accelerates normalization of liver function may be helpful. 

Silymarin, a flavonolignan from *Silybum marianum*, is a complex mixture of four flavonolignan isomers among them silybin is the most active component (7) and is the most used natural compound for the treatment of hepatic diseases worldwide (8, 9).

In animal models silymarin showed protective effect against hepatotoxicity of anti TB drugs (10).

We performed this study among TB cases that experienced DILI to evaluate the potential effectiveness of silymarin in the management of liver dysfunction. 

## Methods

**Study population**

The study was performed at Masih Daneshvari hospital, the national referral center of tuberculosis and lung disease, Shahid Beheshti University of Medical Sciences, Tehran, Iran. In the period of study admitted adult (more than 18 years old) patients with tuberculosis who experienced liver injury caused by standard anti TB regimen were recruited. Pregnant cases, nursing mothers, patients with preexisting liver disease or with concomitant HIV, HBV or HCV infections were excluded. Ethical permission for the study was obtained from the ethics review board of the National Research Institute of Tuberculosis and Lung diseases. Also written informed consent was obtained from all participants. 

**Anti TB regimen**

Standard anti TB regimen was defined as initiation of treatment consisting isoniazid (5 mg/kg), rifampin (10 mg/kg), ethambutol (15 mg/kg) and pyrazinamide (20 mg/kg) as WHO standard (11).

**Diagnosis of drug-induced hepatotoxicity**

DILI was defined as 1) increasing of AST or ALT to three times more than upper limit of normal (40 IU/L), concomitant with symptoms of hepatic toxicity consisting nausea, vomiting, anorexia, weakness and abdominal pain 2) rise in AST or ALT more than five times or total serum bilirubin more than 2 mg/dl (2, 12).

**Study design and interventions**

The investigation was designed as double blind randomized clinical trial, which was registered in the Australian New Zealand Clinical Trial Registry, and the registry number is ACTRN12610000643077.

In the cases of DILI, anti-tuberculosis drugs were stopped and temporarily bridge regimen consisting ethambutol, ofloxacin and Amikacin were initiated. Silymarin containing tablets (Livergol^®^) and its identical placebo were manufactured by Goldaru pharmaceutical company, Isfahan, Iran. Each Livergol^®^ 140 tablet contains dried extract of silybum marianum equivalent to 140 mg silymarin. These patients were randomly allocated into two groups. Group one was received Livergol^®^, three times per day. The second group was received placebo. Drugs and placebo were encoded until analysis of results was done.

Liver function test (LFT) monitoring was being performed three times per week. For both groups intervention was continued for two weeks unless LFT was normalized earlier. 

After normalization of LFT, treatment was continued as American thoracic society (ATS) guideline (12).

**Primary and secondary o** **utcome** **measures**

Primary outcome of study was defined as the time of normalization of liver enzymes and bilirubin. Also severity of DILI, duration of related symptoms, mortality, and hospital stay were compared in two groups. Severity of liver injury was measured by peak values of AST, ALT, total bilirubin and incidence of coagulopathy, hypoglycemia, and encephalopathy. 

As secondary outcome, intervention related adverse effects were compared between placebo and silymarin groups. 

**Table 1:** Basic characteristics of the two groups

Demographics		Silymarin group	Placebo group	p-value
Age*		52±4	57±4	0.40
Sex				1.00
	male female	13 (48.1%) 14 (51.9%)	12 (44.4%) 15 (55.6%)	
Weight (Kg) *		54±2	55±3	0.63
Time of DILI*#		15±3	18±6	0.66
Base LFT*				
	AST ALT Total Bilirubin	33±7 26±5 0.8±0.1	25±2 23±3 0.6±0.1	1.00 0.85 0.22
Initial LFT on DILI*				
	AST ALT Total Bilirubin	197±31 149±21 2.5±0.6	173±32 125±24 3.2±0.9	0.43 0.10 0.80
Total		27	27	

**Statistical analysis**

All data were entered into SPSS (version 15.0) for statistical analysis. A chi-square statistic without Yates’ correction, Fisher’s exact test, the Student’s t-test and the Mann-Whitney U test were used as appropriate. All reported p values are two-sided. A p-value less than 0.05 was considered statistically significant.. 

## Results

**Figure 1:**
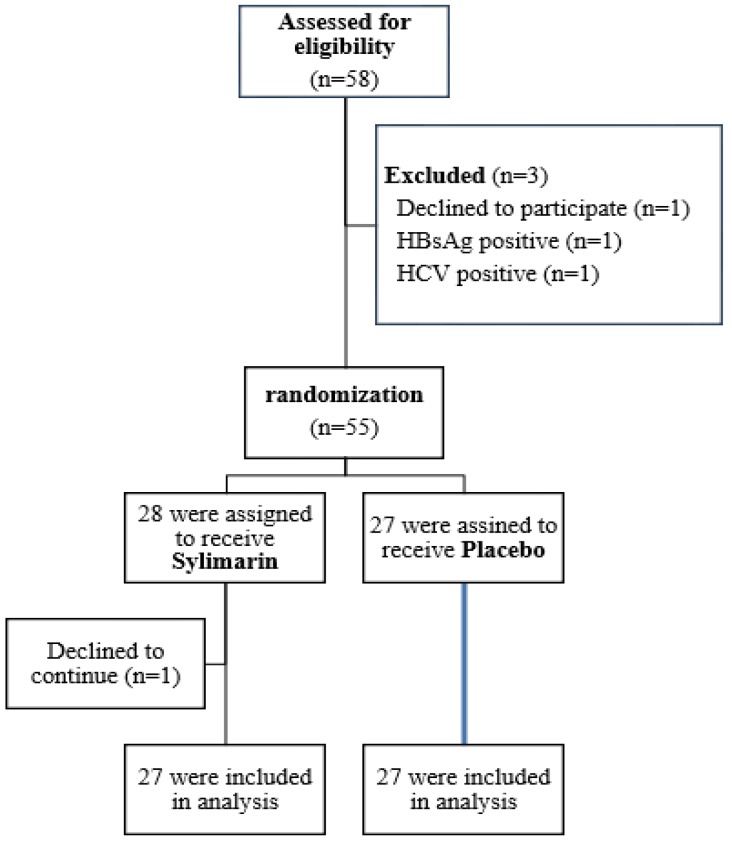
Flow chart of the patients in the study: Enrollment, randomization, and follow-up of the study patients

Abbreviations: HBsAg: Hepatitis B surface antigen, HCV: Hepatitis C virus

Among 58 cases of tuberculosis with DILI, one patient declined to participate in the study and two patients were excluded due to concomitant viral hepatitis. Fifty five cases were eligible, underwent randomization and allocated to either the silymarin group or the placebo group. One patient two days after initiation of intervention declined to continue, so 54 cases were included in the final analysis (Figure 1). Study group consisted 25 men (46.2%) and 29 women with mean age of 54.4 ± 21.6 years. All of the cases had pulmonary tuberculosis. There was not any statistical difference between the two groups concerning sex, age, weight, liver function tests at the beginning of TB treatment and diagnosis of DILI, and interval between initiation of anti TB and DILI (Table 1).

**Table 2:** Comparison of characteristics of liver injury and outcome between two groups

Variables		Silymarin group	Placebo group	p-value
Duration of DILI*#		9±1	8±2	0.06
Peak levels of LFT*				
	AST ALT Total Bilirubin	222±36 182±34 2.7±0.6	211±46 176±38 3.3±0.9	0.54 0.35 0.98
Symptoms duration*¥ (days)				
	Fatigue Anorexia Nausea	3±1 2±1 2±1	2±1 2±1 2±1	.14 0.35 0.60
Hepatic encephalopathy		1 (3.7%)	0 (0%)	1.00
Coagulopathy		1 (3.7%)	0 (0%)	1.00
Hypoglycemia		0	0	-
Death		3 (11.1%)	2 (7.4%)	1.00
Hospital stay (days)*		16±1	16±1	0.29
Total		27	27	

Abbreviations: DILI: Drug induced liver injury, LFT: Liver function tests; AST: Aspartate aminotransferase; ALT: Alanine transaminase

* : Mean±SD;

# : The days elapsed from the emergence of DILI till the liver enzymes became normalized.

¥ : Duration of DILI related symptoms.

**Table 3:** Adverse effects reported by the patients of the two groups

Adverse effects	Silymarin group	Placebo group	p-value
Nausea	2 (7.4%)	4 (14.8%)	0.66
Vomiting	1 (3.7%)	3 (11.1%)	0.61
Diarrhea	0 (0%)	1 (3.7%)	1.00
Pruritus	1 (3.7%)	0 (0%)	1.00
Any side effect*	3 (11.1%)	4 (14.8%)	1.00
Total	27	27	

* Nausea, vomiting, diarrhea and pruritus

As primary outcome, characteristics of liver injury were compared. Duration and severity of DILI were similar between patients who received silymarin and those who received placebo. All events of deaths were TB related and there were no differences concerning all-cause mortality and duration of hospital stay between the two groups (Table 2). Among ten patients, normalization of liver enzymes lasted more than 15 days (no difference between placebo and silymarin groups).

We compared adverse effects possibly related to intervention between two groups. No patient experienced any severe or life threatening adverse effect. One patient in the silymarin group stopped taking the medication despite no reaction to the drug. There was no statistically significant difference in the rate of adverse effects between silymarin and placebo groups. These data are summarized in table 3.

## Discussion

In this double blind randomized clinical trial involving 58 cases of tuberculosis with DILI, no therapeutic effect was observed for silymarin to ameliorate hepatic adverse effects of anti-tuberculosis drugs. Although it was safe, silymarin neither decreased the severity of DILI, nor shortened the duration necessary for liver function normalization, and hospital stay. 

The exact mechanism of anti TB induced DILI is unknown (13). Isoniazid-induced hepatotoxicity is considered idiocyncratic and is probably induced by toxic metabolites. However, the mechanisms of rifampin or pyrazinamide-induced toxicities are unknown (2). Genetic factors especially polymorphisms in drug-metabolizing enzymes may be important (14).

Potential effect of silymarin on the management of hepatic adverse effects of anti-tuberculosis drugs has been investigated by previous studies. The studies were performed in vitro or experimental animals and showed promising results (10, 15, 16), human studies were conflicting. Luangchosiri and colleagues in a double-blind randomized placebo-controlled trial in 55 patients showed the benefit of silymarin to reduce incidence of DILI. They attributed the lower incidence of DILI to a smaller decline in superoxide dismutase levels in the silymarin-treated patients in comparison with controls (17). On the other hand, trials were performed by Gu *et al.* (prospective, multicenter randomized trial) (18) Heo and colleagues (double blind randomized clinical trial) (19) and our team (double blind randomized clinical trial) (20) on 568, 121 and 70 cases, respectively showed no significant hepatoprotective effect of silymarin, concerning reduction of hepatotoxicity, among the patients under tuberculosis treatment. 

Another study was performed by Zhang and colleagues on 183 cases as experimental and 187 cases as control groups. The experimental group received anti-tuberculosis therapy plus silymarin and the control group received anti-tuberculosis therapy plus vitamin C tablets. They found no significant preventive effect for silymarin even there was a potential risk of liver damage (21). 

To our knowledge, our study is the first trial concerning the effect of silymarin to improve DILI caused by anti-tuberculosis drugs. Silymarin did not reduce duration and severity of DILI. It may be due to the different mechanisms responsible for DILI related to anti TB and hepatoprotective effects of silymarin.

Our study had two limitations. First, it was performed in a referral center. Our patients may have experienced more severe disease or more severe DILI. So the results in the field may be different. The second issue is exclusion of patients with preexisting liver disease or concomitant HIV, HBV or HCV infections. We cannot generalize our results to these subgroups of TB patients.

This double blind randomized clinical trial showed that silymarin does not have significant effect in amelioration of hepatic toxicity of anti-tuberculosis drugs. Although silymarin was safe in our study, its usage was not effective to reduce DILI severity, normalization time of liver enzymes and duration of hospital stay. Additional studies are necessary to evaluate effect of silymarin among special high risk groups with DILI
